# SAROS: A dataset for whole-body region and organ segmentation in CT imaging

**DOI:** 10.1038/s41597-024-03337-6

**Published:** 2024-05-10

**Authors:** Sven Koitka, Giulia Baldini, Lennard Kroll, Natalie van Landeghem, Olivia B. Pollok, Johannes Haubold, Obioma Pelka, Moon Kim, Jens Kleesiek, Felix Nensa, René Hosch

**Affiliations:** 1 Institute of Interventional and Diagnostic Radiology and Neuroradiology, University Hospital Essen, Essen, Germany; 2 Institute for Artificial Intelligence in Medicine, University Hospital Essen, Essen, Germany; 3 Data Integration Center, Central IT Department, University Hospital Essen, Essen, Germany

**Keywords:** Anatomy, Machine learning, Data publication and archiving, Computed tomography

## Abstract

The Sparsely Annotated Region and Organ Segmentation (SAROS) dataset was created using data from The Cancer Imaging Archive (TCIA) to provide a large open-access CT dataset with high-quality annotations of body landmarks. In-house segmentation models were employed to generate annotation proposals on randomly selected cases from TCIA. The dataset includes 13 semantic body region labels (abdominal/thoracic cavity, bones, brain, breast implant, mediastinum, muscle, parotid/submandibular/thyroid glands, pericardium, spinal cord, subcutaneous tissue) and six body part labels (left/right arm/leg, head, torso). Case selection was based on the DICOM series description, gender, and imaging protocol, resulting in 882 patients (438 female) for a total of 900 CTs. Manual review and correction of proposals were conducted in a continuous quality control cycle. Only every fifth axial slice was annotated, yielding 20150 annotated slices from 28 data collections. For the reproducibility on downstream tasks, five cross-validation folds and a test set were pre-defined. The SAROS dataset serves as an open-access resource for training and evaluating novel segmentation models, covering various scanner vendors and diseases.

## Background & Summary

Medical imaging plays a crucial role in the diagnosis and treatment of various diseases. Computed tomography (CT) imaging is one of the most commonly used imaging modalities, allowing for detailed visualization of internal organs and structures. In recent years, the use of CT imaging for body composition analysis (BCA) has gained increasing attention in clinical research. However, developing accurate and efficient segmentation algorithms for CT images remains a challenging task, mainly due to the limited availability of publicly accessible datasets with high-quality annotations.

Many published works on BCA were conducted on in-house data^1–4^ and kept their annotations private or considered only a very specific part of the body^5^. Commonly used locations for manual body composition assessments in the clinical routine are the C3^5,6^ and L3^7–12^ vertebrae, which implies that only single slices in the abdomen and head/neck are measured. Recently published datasets have expanded the pool of data and labels available, including different annotated anatomical landmarks and structures. The TotalSegmentator dataset^13–16^ provided segmentations for 117 anatomical structures such as organs, vessels, and specific bones and muscles. CT Volumes with Multiple Organ Segmentations (CT-ORG)^17^ offered segmentations of some organs and a large-scale segmentation of the bones. AbdomenCT-1K targeted segmentations of four abdominal organs, while the Whole Abdominal Organ Dataset (WORD)^18^ published segmentations of 16 abdominal organs. Moreover, coding challenges also contributed to the rising number of datasets. The Liver Tumor Segmentation Benchmark (LiTS)^19^ and the Kidney Tumor Segmentation Challenge (KiTS)^20,21^ focused on annotations for liver and kidney tumors, respectively. The Lung CT Segmentation Challenge (LCTSC)^22–24^ provided thoracic organs and spinal cord segmentations, while the aim of the Lung Nodule Analysis Challenge 2016 (LUNA16)^25,26^ was the segmentation of lung lobes and nodules. The Combined Healthy Abdominal Organ Segmentation (CHAOS)^27,28^ and the Multi-Modality Abdominal Multi-Organ Segmentation Challenge 2022 (AMOS22)^29^ both provided abdominal organ segmentations for CT and Magnetic Resonance (MR) imaging. The Fast and Low-Resource Semi-supervised Abdominal Organ Segmentation 2022 (FLARE22)^30,31^ also focused on abdominal organs but used semi-supervised learning for fast and low-resource segmentation. FLARE23^32^ extended this concept by adding tumor segmentation in these abdominal regions. Additionally, the Head and Neck Autosegmentation challenge^33^ used the Public Domain Database for Computational Anatomy (PDDCA) segmentations for small head and neck organs and bones.

In most datasets, the focus of the annotation is primarily on organs or pathologies, leaving the rest of the body unexplained. For example, the muscle segmentations from the TotalSegmentator dataset do not cover all the muscle groups of the body. Therefore, the focus of these annotations is different and hardly usable for deriving BCA biomarkers. Furthermore, other structures such as the abdominal cavity, the subcutaneous tissue, and the mediastinum are not publicly available.

In this work, we present the *Sparsely Annotated Region and Organ Segmentation (SAROS)* dataset using publicly available data from The Cancer Imaging Archive (TCIA)^22^. The goal of this dataset is to provide a large open-access annotated CT dataset for building automated BCA pipelines for the whole body^3,4^. In contrast to other openly available datasets, this dataset provides large-scale annotation of body regions, including the subcutaneous tissue, all muscles and bones, the abdominal and thoracic cavities, the mediastinum, and the pericardium. Abdominal, thoracic, and head and neck organs such as liver, lungs, or esophagus were not included, since they are already available in many publicly available imaging datasets. However, the dataset offers annotations of smaller organs such as the thyroid, submandibular and parotid glands. Furthermore, SAROS provides segmentations of the breast implant, which is valuable for improving the differentiation of subcutaneous tissue and could also contribute to more accurate diagnoses of breast pathologies. Additionally, the dataset also includes segmentations of the brain and spinal cord, as well as the segmentation of body parts such as the head, torso, and left/right arms and legs. Another difference with existing datasets is that the SAROS segmentations collectively cover all body voxels. Interested readers can find more information on the applicability of these segmentations for BCA in the Technical Validation section.

**Fig. 1:**
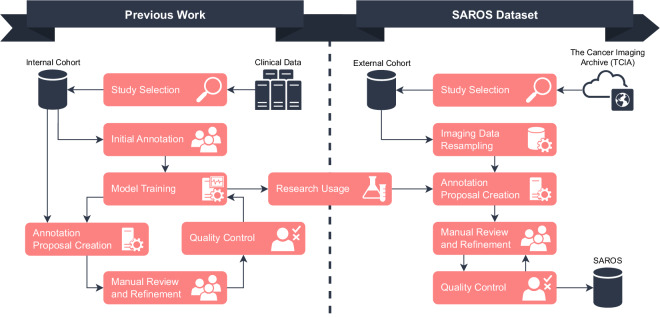
Visualization of the dataset creation process. On the left side, the dataset creation of the internal dataset is shown, whereas on the right side, the transfer of existing segmentation models for the SAROS creation is shown.

The dataset consists of 900 CTs, split into five pre-defined cross-validation folds and a test set, each consisting of 150 CTs. In total, 20,157 slices were annotated with two different label sets. The dataset creation process as well as the relationship to prior work^3^ is shown in Fig. 1. SAROS was gathered from TCIA data sources, annotation candidates were generated using existing in-house BCA models, manual revision was conducted, and manual and automatic quality control was performed.

## Methods

### Study population and data conversion

The cohort used in SAROS was built using available imaging data from various collections of TCIA. All available image series on TCIA were searched for keywords in the series description as well as metadata in DICOM by using their REST API. Those keywords were used to restrict the scan region, e.g. lung, thorax, abdomen, or whole-body. In addition, only imaging series with a soft-tissue reconstruction kernel were included. Furthermore, terms for the selection of the contrast agent phases such as arterial or portal-venous were also used. Three groups of imaging series were created based on the captured body region: abdominal, thoracic, and whole-body. Scans from the abdomen and thorax group were mostly contrast-enhanced, whereas scans from the whole-body group were mostly non-contrast-enhanced. For each body region, a total of 300 CTs were collected, resulting in 300 patients with abdominal scans (150 female), 283 patients with thoracic scans (138 female), and 299 patients with whole-body scans (150 female). Ethical approval was not required for this study, as it exclusively utilizes data already available on TCIA. In collaboration with TCIA, a new collection was established, based on previously published data but enriched with new segmentations.

In total, 900 CTs were compiled from 28 TCIA data collections. A detailed overview of how many CTs were drawn from each data collection and for each group is shown in Table 1. Abdomen and thorax scans were easily acquired from 6 and 5 data collections, respectively, while whole-body scans were rarer and thus scattered across 22 data collections. Still, most of the whole-body scans are truncated at the upper legs. As previously mentioned, the CTs were selected randomly across all search results and there was no attempt to reduce the number of required data collections.

**Table 1:** Number of CTs from data collections on TCIA used for SAROS and whether the collection has restricted access or not.

Collection	Abdomen	Thorax	Whole-body	Restricted
ACRIN-FLT-Breast^50,51^	0	0	32	No
ACRIN-HNSCC-FDG-PET-CT^52,53^	0	0	48	Yes
ACRIN-NSCLC-FDG-PET^54,55^	0	78	51	No
Anti-PD-1_Lung^56^	0	0	12	No
Anti-PD-1_Melanoma^57^	0	0	2	Yes
C4KC-KiTS^20,21^	175	0	0	No
COVID-19-NY-SBU^58^	0	0	1	No
CPTAC-CM^59^	0	0	1	No
CPTAC-LSCC^60^	0	0	3	No
CPTAC-LUAD^61^	0	0	1	No
CPTAC-PDA^62^	8	0	0	No
CPTAC-UCEC^63^	25	0	1	No
HNSCC^64–66^	0	0	17	Yes
Head-Neck Cetuximab^67,68^	0	0	12	Yes
LIDC-IDRI^69,70^	0	133	0	No
Lung-PET-CT-Dx^71^	0	15	2	No
NSCLC Radiogenomics^72–75^	0	0	7	No
NSCLC-Radiomics^76,77^	0	56	0	No
NSCLC-Radiomics-Genomics^77,78^	0	18	2	No
Pancreas CT^79,80^	58	0	0	No
QIN-HEADNECK^81,82^	0	0	94	Yes
Soft-tissue-Sarcoma^83,84^	0	0	6	Yes
TCGA-HNSC^85^	0	0	1	Yes
TCGA-LIHC^86^	32	0	1	Yes
TCGA-LUAD^87^	0	0	2	Yes
TCGA-LUSC^88^	0	0	3	Yes
TCGA-STAD^89^	2	0	0	Yes
TCGA-UCEC^90^	0	0	1	Yes
=Number of CTs	300	300	300	
=Number of Collections	6	5	22	

The downloaded DICOM data from TCIA was converted to the Neuroimaging Informatics Technology Initiative (NIfTI)^34^ format using the SimpleITK^35^ library.

### Segmentation

**Fig. 2:**
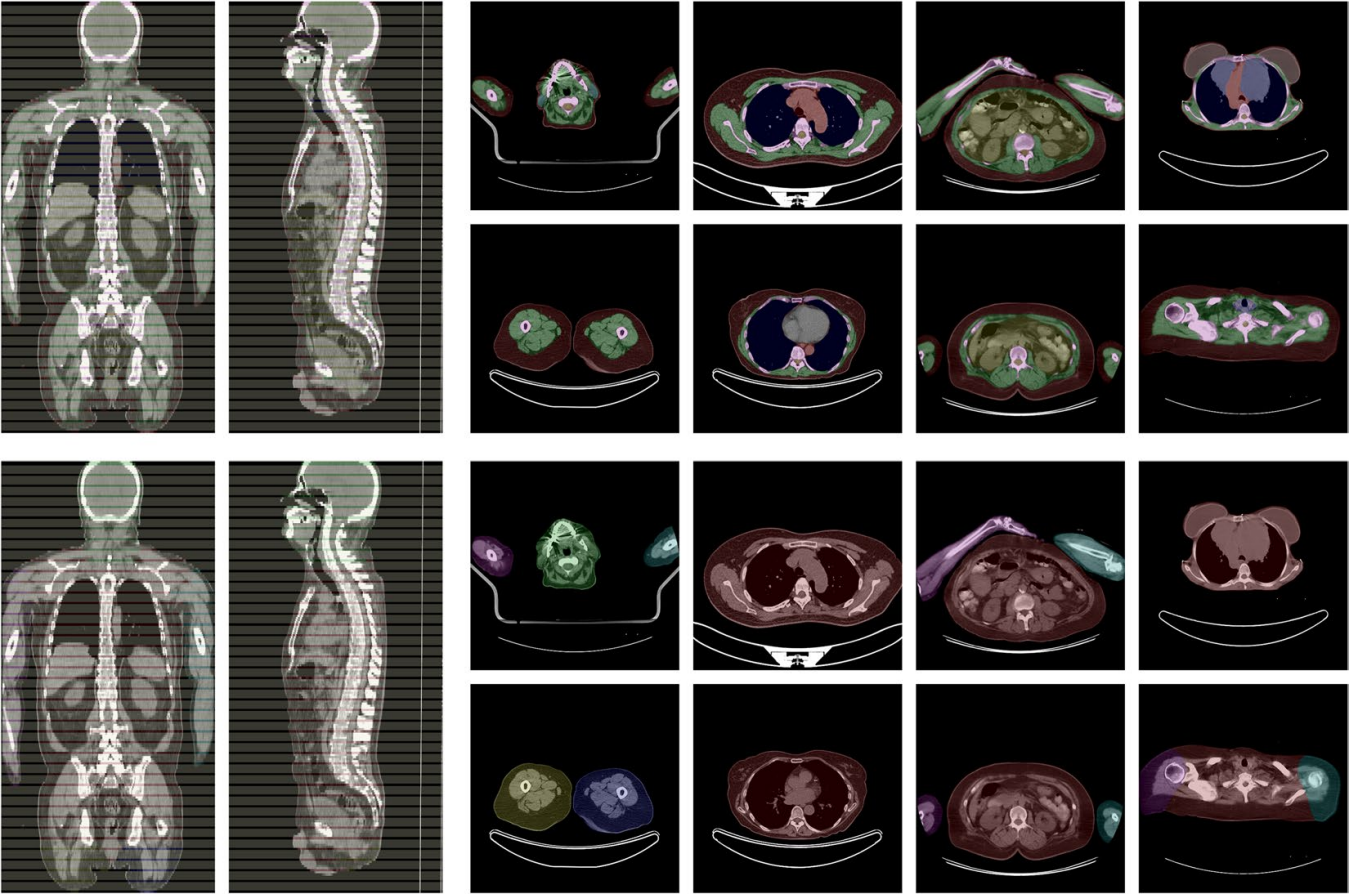
Exemplary slices of the annotated CT cases. On the left side, the sparse annotation scheme is visible in the coronal and sagittal slices, since only every fifth axial slice was annotated. On the top, the body regions label set is shown (e.g. red = subcutaneous tissue; green = muscles; blue = thoracic cavity; yellow = abdominal cavity), on the bottom, the corresponding body parts label set is shown (red = torso; yellow = right leg; blue = left leg; green = head; pink = right arm; cyan = left arm).

SAROS complements the imaging data with two segmentation files. First, the body-region segmentation represents anatomical structures in the body, e.g. abdominal cavity, muscles, pericardium, and many more. Second, the body-parts segmentation represents a coarse grouping of parts of the body, namely both arms and legs, head, and torso. Exemplary slices from annotated cases can be found in Fig. 2.

In previous work, an internal dataset from data in the clinical routine was created to train deep learning segmentation networks to enable fully automated body composition analysis^3^. This internal dataset initially covered only contrast-enhanced abdominal scans but was extended over time to also support whole-body CTs, contrast-enhanced and non-contrast-enhanced CTs, as well as more semantic regions in order to extract additional biomarkers and to increase the stability of the model predictions^3^. The internal dataset can be requested from the University Hospital Essen by sending an email with the subject “Request: access to Internal BCA/SAROS dataset” to the corresponding researcher René Hosch (rene.hosch@uk-essen.de) at the Institute for Artificial Intelligence in Medicine (IKIM) with a short summary of the intended use of the dataset. However, as the dataset contains private patient data, it cannot be made publicly available for legal reasons and access can only be granted on specific request. It should also be noted that the internal dataset and the model based on it were only used as a fundament for the initial annotation of the SAROS dataset. Thus, the SAROS dataset provides an independent and larger manually segmented dataset than the internal dataset.

The body-region segmentation in SAROS was annotated with the help of existing models trained on the internal dataset. For this purpose, the image data was resampled to 5 mm slice thickness, as these models are based on 3D network architectures and were trained on 5 mm CTs. In each CT, an annotation for every fifth slice was proposed, which was reviewed and refined by a human reader using ITK Snap^36^. Every other slice was set to an ignore label (numeric value 255), which resulted in a sparse annotation of the CT scans. This approach drastically reduced the required annotation time compared to starting from scratch, since many proposed contours were already precise enough, and only regions with high uncertainty had to be refined. For example, partial volume effects in 5 mm CTs or abnormal anatomy are a common source of errors for the decision boundaries. In case of strong beam hardening artifacts, mainly in the region of dentition or pelvis due to metallic implants, the slices in question were set completely to an ignore label by the human reviewer. After reviewing and refining all proposed annotations, a quality control team ensured the validity of the annotations and reiterated the refinement process if required. On the one hand, manual control by experienced reviewers was done to identify annotation errors caused by lack of experience or imprecise label definition. On the other hand, an automatic approach was used to identify errors. More details on this are available in the Technical Validation section.

For the body-part segmentation, the refined and reviewed annotations were used to generate a body mask with pre-annotated part labels using simple rules on the body-regions segmentation and a connected component analysis. Suppose that a slice is below the abdominal cavity and has exactly two connected components, it can easily be assumed that those components correspond to the left and right leg. Similarly, a slice showing the abdominal or thoracic cavity with two or three connected components most likely also shows the left and/or right arm. Thus the annotation effort was again greatly reduced, since only a few slices had to be modified where an automatic derivation was not possible.

### Split definition

In order to foster reproducibility, five cross-validation folds as well as a test set were predefined, consisting of 150 samples each. Users who want to use a train/validation/test approach without cross-validation, can use “fold-1” for validation and the union of “fold-2”, “fold-3”, “fold-4”, and “fold-5” for training (see Data Records section). Since only 18 CTs in total contain a breast implant label, these were evenly distributed using stratified random sampling. The remaining cases were randomly assigned to the six groups, ensuring that CTs from the same patient were assigned to the same split.

## Data Records

The annotated data is stored at TCIA in the collection “SAROS - A large, heterogeneous, and sparsely annotated segmentation dataset on CT imaging data (SAROS)”^22,37^. The segmentations for each CT are stored in a separate directory with the name “case_xxx” (“xxx” is a zero-padded three-digit case index starting with zero), where the body regions segmentation file “body-regions.nii.gz” and the body parts segmentation file “body-parts.nii.gz” are available. The segmentation files always contain fully annotated axial slices or completely ignored axial slices (numeric value 255). Table 2 contains both label sets (body regions and body parts) including the enumeration index corresponding to the numerical value of the respective label.

**Table 2:** List of available labels within the body-regions and body-parts segmentation files including the corresponding label ID in the segmentation NIFTI files.

Label ID	“body-regions.nii.gz”	“body-parts.nii.gz”
**0**	Background	Background
**1**	Subcutaneous Tissue	Torso
**2**	Muscle	Head
**3**	Abdominal Cavity	Right Leg
**4**	Thoracic Cavity	Left Leg
**5**	Bones	Arm Right
**6**	Parotid Glands	Arm Left
**7**	Pericardium	—
**8**	Breast Implant	—
**9**	Mediastinum	—
**10**	Brain	—
**11**	Spinal Cord	—
**12**	Thyroid Glands	—
**13**	Submandibular Glands	—

A comma-separated value (CSV) file, named “Segmentation Info”, is provided with information about each case, e.g. information about the TCIA identifiers, gender, and CT orientation. Additionally, the column “split” states if the case belongs to one of the five pre-defined cross-validation folds (“fold-1”, …, “fold-5”) or the test set (“test”).

## Technical Validation

During the annotation process, a quality control team consisting of senior annotators, a data scientist, and a senior radiologist with 7 years of experience in abdominal imaging, refined and reviewed segmentations first manually and afterward automatically. Manual quality control was mainly based on the experience of the human readers and the internal annotation guidelines. The automatic quality control implemented a few test cases regarding the presence or absence of labels, unusual instance counts for a specific label or neighbor label constraints. For instance, in the abdominal cavity region, the existing models sometimes made tiny mistakes in vessels with high density, due to calcifications or the presence of a contrast agent, and predicted it to be bone. Those errors were sometimes missed by human readers and thus a 2D connected component analysis was performed in order to identify potential instances with invalid neighbors. Additionally, it was ensured that both annotations, “body-parts.nii.gz” and “body-regions.nii.gz”, match pixel-perfect regarding background and foreground labels.

In Table 3, label statistics are shown for each cross-validation fold, the test set, and the overall dataset. In total 900 CTs were annotated, each split contains 150 CTs, and breast implant labels are evenly distributed among all splits.

**Table 3:** Label statistics for each cross-validation fold, test split, and the overall dataset.

	Fold-1	Fold-2	Fold-3	Fold-4	Fold-5	Test	Overall
Abdominal Cavity	150 / 1838	150 / 1839	150 / 1802	150 / 1759	150 / 1863	150 / 1855	900 / 10956
Background	150 / 3360	150 / 3429	150 / 3278	150 / 3268	150 / 3420	150 / 3395	900 / 20150
Bones	150 / 3333	150 / 3407	150 / 3267	150 / 3249	150 / 3405	150 / 3374	900 / 20035
Brain	41 / 186	43 / 206	44 / 191	45 / 210	47 / 213	42 / 181	262 / 1187
Breast Implant	3 / 11	3 / 16	3 / 12	3 / 12	3 / 16	3 / 16	18 / 83
Mediastinum	150 / 1185	150 / 1202	150 / 1204	150 / 1196	150 / 1197	150 / 1213	900 / 7197
Muscle	150 / 3327	150 / 3396	150 / 3262	150 / 3242	150 / 3398	150 / 3367	900 / 19992
Parotid Glands	48 / 106	45 / 100	45 / 91	45 / 98	48 / 109	43 / 96	274 / 600
Pericardium	143 / 576	148 / 594	148 / 588	149 / 593	149 / 583	147 / 588	884 / 3522
Spinal Cord	150 / 2552	150 / 2558	150 / 2526	150 / 2506	150 / 2561	150 / 2584	900 / 15287
Subcutaneous Tissue	150 / 3335	150 / 3407	150 / 3267	150 / 3249	150 / 3404	150 / 3374	900 / 20036
Submandibular Glands	49 / 67	46 / 56	47 / 61	46 / 62	44 / 59	43 / 57	275 / 362
Thoracic Cavity	150 / 1386	150 / 1416	150 / 1417	150 / 1412	150 / 1420	150 / 1415	900 / 8466
Thyroid Glands	98 / 166	100 / 186	96 / 167	96 / 176	98 / 173	99 / 195	587 / 1063
Arm Left	98 / 941	100 / 1028	97 / 1029	99 / 928	100 / 997	99 / 1011	593 / 5934
Arm Right	100 / 969	98 / 1033	98 / 1000	98 / 929	99 / 981	99 / 967	592 / 5879
Background	150 / 3360	150 / 3429	150 / 3278	150 / 3268	150 / 3420	150 / 3395	900 / 20150
Head	75 / 414	81 / 437	73 / 407	79 / 434	88 / 465	78 / 413	474 / 2570
Leg Left	65 / 283	68 / 324	63 / 248	58 / 240	70 / 277	70 / 274	394 / 1646
Leg Right	65 / 284	68 / 322	63 / 247	59 / 240	71 / 279	71 / 273	397 / 1645
Torso	150 / 2848	150 / 2860	150 / 2820	150 / 2764	150 / 2899	150 / 2905	900 / 17096

For each label, the number of CTs as well as the number of slices are stated, on which the label is present. Statistics are reported for both label sets: (top) body regions and (bottom) body parts.

### Baseline models

Two baseline models were trained to provide benchmarkss for the segmentation of body parts and body regions for the SAROS dataset. The nnUNet^16^ framework was chosen for this purpose, as it is auto-configuring and requires little adjustment. The models were trained using a 2D nnUNet with 5-fold cross validation using the splits outlined in the “Data Records” section. The scripts used for this purpose are available on GitHub (https://github.com/UMEssen/saros-dataset/tree/main/training). The README file in the repository provides guidelines for training and evaluation of the models using the Dice score^38^ and the Normalized Surface Dice (NSD)^39^. The NSD calculates the frequency at which the surface distance between volumes measures under 3 mm, a metric previously utilized by TotalSegmentator^13^. The scores were calculated as the average of the single labels over the CT scans and are presented in Table 4 for both models.

**Table 4:** Evaluation of two 2D nnUNet models trained on the SAROS dataset.

Body Regions	Dice	NSD	Body Parts	Dice	NSD
Abdominal Cavity	0.988	0.997	Arm Left	0.856	0.889
Bones	0.974	0.996	Arm Right	0.868	0.905
Brain	0.991	0.997	Head	0.739	0.764
Breast Implant	0.585	0.599	Leg Left	0.942	0.945
Mediastinum	0.906	0.957	Leg Right	0.954	0.957
Muscle	0.971	0.994	Torso	0.992	0.995
Parotid Glands	0.762	0.848			
Pericardium	0.966	0.985			
Spinal Cord	0.955	0.986			
Subcutaneous Tissue	0.98	0.995			
Submandibular Glands	0.56	0.651			
Thoracic Cavity	0.988	0.997			
Thyroid Glands	0.825	0.931			
**Average**	**0.881**	**0.918**		**0.892**	**0.909**

The models were evaluated using the Dice and the Normalized Surface Dice (NSD) scores. The scores were computed as the average of each label over the CT scans. Both scores range from 0 to 1, where 0 is the worst and 1 is the best possible score.

### Further discussion of methods

Datasets similar to SAROS have already been successfully used for building a BCA pipeline for multiple research studies^3,4,40–43^. However, none of these datasets were released open-source.

**Fig. 3:**
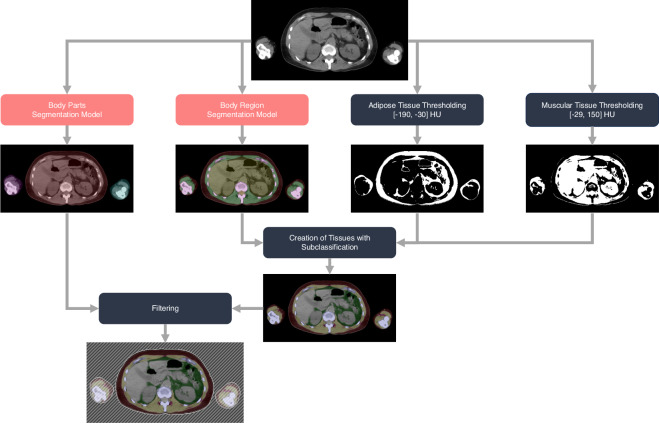
Exemplary workflow of a body composition analysis pipeline using segmentation models trained on the SAROS dataset.

An exemplary pipeline for BCA from these studies is presented in Fig. 3. First, the “body-regions” segmentation is used to predict semantic regions. The regions can then be subclassified using Hounsfield Unit (HU) thresholding to distinguish the localization of adipose tissue^7,44–46^ and to refine the muscle segmentation^11^. Both the muscular and the adipose tissue can be derived based on known and established HU thresholds (adipose tissue −190 to −30 HU, muscular tissue −29 to 150 HU)^47^, or could be computed by material decomposition algorithms using either dual-energy CTs (DECT) or photon-counting CTs (PCCT)^48,49^. In research projects, it has been shown that especially ratios between bone, muscular tissue, and different adipose tissue types are important biomarkers for diagnostic and prognostic endpoints^40–43^.

## Usage Notes

The segmentations and the CSV file provided in the SAROS TCIA collection^22,37^ are freely accessible and can be downloaded from the TCIA collection page. However, the underlying CT images originate from a combination of datasets, all of which require the user to have a registered TCIA account. Moreover, some datasets require the user to sign a TCIA Data Usage Agreement. A source code repository is available on Github (https://github.com/UMEssen/saros-dataset) with a download.py script to easily download and convert the CT scans to resampled NIfTI images. After running the download.py script, the CT image is downloaded, resampled, and stored as “image.nii.gz”. If the option–save-original-image was provided, the original CT image without resampling is additionally stored as “image-original.nii.gz”.

## Data Availability

A script to download and resample the data is available on GitHub (https://github.com/UMEssen/saros-dataset).
